# Long-term cost-effectiveness of implementing a lifestyle intervention during pregnancy to reduce the incidence of gestational diabetes and type 2 diabetes

**DOI:** 10.1007/s00125-023-05897-5

**Published:** 2023-03-18

**Authors:** Melanie Lloyd, Jedidiah Morton, Helena Teede, Clara Marquina, Dina Abushanab, Dianna J. Magliano, Emily J. Callander, Zanfina Ademi

**Affiliations:** 1 Centre for Medicine Use and Safety, Faculty of Pharmacy and Pharmaceutical Sciences, Monash University, Melbourne, Australia; 2 School of Public Health and Preventive Medicine, Monash University, Melbourne, Australia; 3 Diabetes and Population Health, Baker Heart and Diabetes Institute, Melbourne, Australia

**Keywords:** Cost-effectiveness, Decision modelling, Dietary intervention, Epidemiology, Gestational diabetes mellitus, Life table modelling, Physical activity, Pregnancy, Type 2 diabetes mellitus

## Abstract

**Aims/hypothesis:**

The aim of this study was to determine the long-term cost-effectiveness and return on investment of implementing a structured lifestyle intervention to reduce excessive gestational weight gain and associated incidence of gestational diabetes mellitus (GDM) and type 2 diabetes mellitus.

**Methods:**

A decision-analytic Markov model was used to compare the health and cost-effectiveness outcomes for (1) a structured lifestyle intervention during pregnancy to prevent GDM and subsequent type 2 diabetes; and (2) current usual antenatal care. Life table modelling was used to capture type 2 diabetes morbidity, mortality and quality-adjusted life years over a lifetime horizon for all women giving birth in Australia. Costs incorporated both healthcare and societal perspectives. The intervention effect was derived from published meta-analyses. Deterministic and probabilistic sensitivity analyses were used to capture the impact of uncertainty in the model.

**Results:**

The model projected a 10% reduction in the number of women subsequently diagnosed with type 2 diabetes through implementation of the lifestyle intervention compared with current usual care. The total net incremental cost of intervention was approximately AU$70 million, and the cost savings from the reduction in costs of antenatal care for GDM, birth complications and type 2 diabetes management were approximately AU$85 million. The intervention was dominant (cost-saving) compared with usual care from a healthcare perspective, and returned AU$1.22 (95% CI 0.53, 2.13) per dollar invested. The results were robust to sensitivity analysis, and remained cost-saving or highly cost-effective in each of the scenarios explored.

**Conclusions/interpretation:**

This study demonstrates significant cost savings from implementation of a structured lifestyle intervention during pregnancy, due to a reduction in adverse health outcomes for women during both the perinatal period and over their lifetime.

**Graphical abstract:**

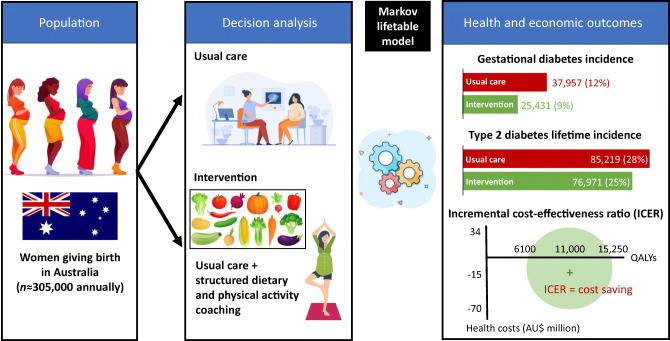

**Supplementary Information:**

The online version contains peer-reviewed but unedited supplementary material available at 10.1007/s00125-023-05897-5.

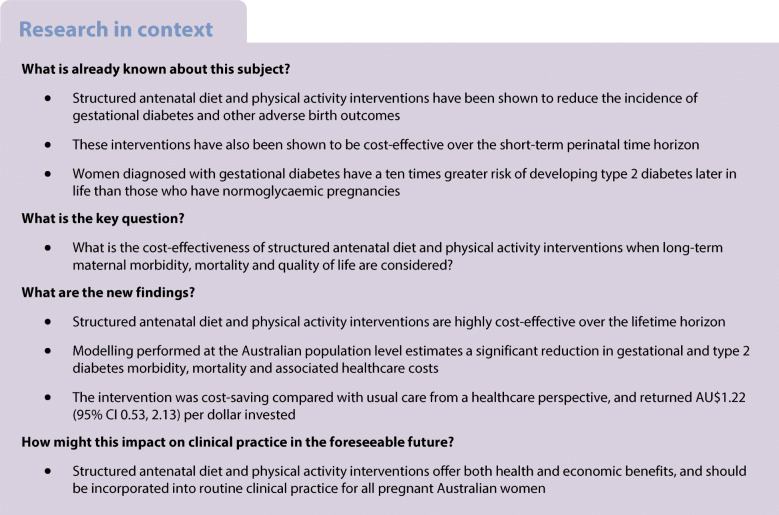



## Introduction

Pregnancy-related weight gain is associated with adverse health outcomes, for both mother and infant, during the perinatal period and beyond. In the perinatal period, these outcomes include the development of gestational diabetes mellitus (GDM), an increased risk of birth intervention, and requirement for neonatal intensive care (NICU) admission [1–3]. In 2015, one in nine pregnant women in Australia was diagnosed with GDM, of whom 44% required induction of labour and 40% had a Caesarean birth [4]. Infants born to mothers with GDM are 80% more likely to require admission to NICU or a special care nursery (SCN), compared to mothers without GDM [4]. GDM has also been linked with adverse health outcomes over the lifespan for the mother, including a tenfold increase in the risk of developing type 2 diabetes [5], the lifetime risk of which is already very high in Australia [6].

Both GDM and type 2 diabetes are responsible for a large cost burden within the Australian health system. Health service costs associated with antenatal care and birth are 13% higher for women with diabetes compared with normoglycaemic pregnancies [7]. In 2015, type 2 diabetes was the 12th largest contributor to Australia’s total disease burden, and AU$577 million was spent managing type 2 diabetes among women with this condition [8]. The chronic nature of the disease, intensive management regimen, and associated health complications significantly affect the wellbeing of sufferers over their lifetime [9, 10]. Interventions that reduce or delay the onset of diabetes therefore have the potential to significantly improve the burden of type 2 diabetes for women and society.

Strong evidence now exists to support the routine implementation of lifestyle interventions for all women during pregnancy to reduce excessive gestational weight gain and the incidence of associated adverse health outcomes [11, 12]. These interventions have been shown to reduce the risk of GDM by over 30% [12]. They have also been shown to be cost-effective over a short time horizon from the perspective of the healthcare system [13–15].

Multiple meta-analyses have provided strong evidence linking GDM with an increased risk of developing type 2 diabetes across the woman’s lifespan [5, 16, 17]. However, few studies have focused on the long-term costs and consequences of lifestyle interventions to reduce the incidence of GDM in pregnant women [18]. As dysglycaemia is a continuum, it is reasonable to assume that a reduction in the incidence of GDM will have an impact on the incidence of type 2 diabetes, particularly in young women. This study aims to determine the lifetime cost-effectiveness and return on investment (ROI) of implementing a structured lifestyle intervention to reduce excessive gestational weight gain and the associated incidence of GDM.

## Methods

### Model structure

A decision-analytic Markov model with one-year cycles was developed to compare the health and economic outcomes for (1) implementation of a structured lifestyle intervention during pregnancy aimed at preventing GDM and subsequently type 2 diabetes; and (2) the current usual antenatal care in Australia (no routine provision of structured lifestyle intervention). In addition to the costs associated with GDM itself, we focused on the excess burden of type 2 diabetes among women with GDM, using life table modelling to capture diabetes morbidity and mortality over a lifetime horizon, from both healthcare and societal perspectives. The lifetime horizon was chosen due to the chronic lifelong impact of type 2 diabetes on morbidity, mortality and quality of life.

**Fig. 1:**
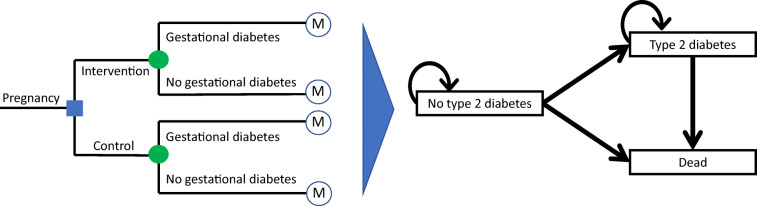
Diagrammatic illustration of the Markov model used in combination with decision analysis. The blue square indicates a decision node; the green circles indicate a chance node (with transition probabilities determined by available evidence); the circles containing ‘M’ indicate the Markov node where participants enter the Markov process

The decision model started at conception, and captured costs and outcomes associated with (1) GDM; and (2) no GDM antenatal health states. After giving birth, participants enter a Markov model to capture ongoing health states: (1) ‘no type 2 diabetes’, (2) ‘type 2 diabetes’; and (3) ‘death’ (Fig. 1). All individuals enter the Markov process in the ‘no type 2 diabetes’ state at birth, and for each model cycle stay in this state or progress to ‘type 2 diabetes’ or ‘dead’. The main outcome was cost per quality-adjusted life year (QALY) gained, expressed as an incremental cost-effectiveness ratio (ICER). A discounting rate of 5% was applied in the model as per Australian guidelines [19], and a cost-effectiveness threshold of AU$50,000/QALY was assumed.

### Population of interest

Pregnant Australian women aged between 15 and 49 years and giving birth in hospital were included in the analysis. Pregnant women were grouped into age categories (15–19, 20–24, 25–29, 30–34, 35–39, 40–44 and 45–49 years) and followed up until death or they reached 85 years of age. Each cohort was modelled separately to allow for age-related differences in the number of pregnant women and the prevalence of GDM and type 2 diabetes (see electronic supplementary material [ESM] Table 1).

Pregnant women with type 1 diabetes or type 2 diabetes diagnosed pre-conception were excluded from the base case analysis (approximately 1% of pregnant Australian women), with prevalence according to age group taken from the Australian Perinatal Dataset (2016) [20]. The risk of death during pregnancy was not incorporated into the model due to the extremely low maternal death rate in Australia (6.4 per 100,000 births) [21].

### Transition probabilities under usual care

The prevalence of GDM for each age group was taken from the Australian Institute of Health and Welfare Gestational Diabetes in Australia Report 2015–2016 [4]. For the transition between ‘no GDM’ to ‘type 2 diabetes’, the age and female-specific incidence of type 2 diabetes in 2016 were obtained from the National Diabetes Services Scheme (NDSS) (ESM Table 2) [22].

We assumed that the RR for progression to type 2 diabetes after pregnancy was 9.51 for women with GDM compared with normoglycaemic pregnant women; this number was derived from a published meta-analysis (see Table 1) [5]. This meta-analysis found no evidence of interaction of maternal age and GDM with the risk of subsequently developing type 2 diabetes; thus, we did not adjust this RR for age. As the RR is calculated as the incidence of type 2 diabetes in the group with GDM divided by the incidence in the group without GDM, but the incidence rate obtained from the NDSS included both these groups, a correction factor was applied to the RR using a method described previously [23]. The equation for adjusted RR [23] is RR_adjusted_ = RR_original_/[(GDM_prevalence_ × RR_original_) + (1 – GDM_prevalence_)], where RR_original_ is the risk in the group with GDM divided by the risk in the group without GDM, and GDM_prevalence_ refers to the prevalence of GDM in the total Australian female population aged 15–49 years (0.65%) [20]. This produces an adjusted RR that is multiplied by the type 2 diabetes incidence in the total population to determine the type 2 diabetes incidence in the GDM group. This is then subtracted from the total population incidence to determine the incidence in the group without GDM. We used a GDM prevalence rate of 0.65% for the total Australian female population (aged 15–49 years) in this calculation [20]. It was assumed that the risk of progression to type 2 diabetes was the same for pregnant women without GDM and non-pregnant women. The equation used to calculate the adjusted RR is indicated below Table 1, and produced an adjusted RR for the relative risk of progression to type 2 diabetes after pregnancy of 9.01. As with all recent studies of GDM prevalence, changing diagnostic criteria are an important consideration, specifically implementation of the International Association of Diabetes in Pregnancy Study Group (IADPSG) criteria from 2017 onwards. All population-level data used in the model were therefore obtained from 2016 to avoid the issue of use of new diagnostic criteria based on pregnancy complications, rather than type 2 diabetes risk [24], contaminating the analysis. Only one study used in the meta-analysis described above used the IADPSG GDM diagnostic criteria (assigned a 0.93% weighting factor) [5], so we can be confident that the RR applied in the model has not been contaminated by implementation of the updated criteria.

**Table 1:** Base case input parameters and their distributions

Input parameter	Mean	Range^a^		Distribution	Reference
		Lower	Upper		
Utility weights					
Age group (years)					
18–24	0.95	0.92	0.98	Beta	McCaffrey et al [26]
25–34	0.95	0.90	0.99	Beta	
35–44	0.91	0.86	0.96	Beta	
45–54	0.87	0.81	0.93	Beta	
55–64	0.88	0.82	0.94	Beta	
65–74	0.87	0.80	0.94	Beta	
≥ 75	0.82	0.74	0.90	Beta	
Type 2 diabetes population	0.79	0.68	0.89	Beta	Beaudet et al [27]
RR of progression to T2DM^b^	9.51 (9.01)	7.14 (6.86)	12.67 (11.77)	Log normal	Vounzoulaki et al [5]
RR for developing GDM^c^					
Base case (diet and/or PA)	0.67	0.58	0.77	Log normal	Teede et al [12], Lloyd et al [15]
Diet only	0.66	0.50	0.85	Log normal	
PA only	0.62	0.50	0.77	Log normal	
Diet and PA	0.75	0.58	0.97	Log normal	

Utility weights shown are for the Australian female population. Population demographics, disease prevalence and cost parameters and distributions are provided in ESM Tables 1–5

^a^Range applied in the deterministic sensitivity analysis

^b^RR of progression to type 2 diabetes after GDM vs normoglycaemic pregnancy (adjusted). The value in parentheses is the adjusted RR. RR_adjusted_ may be multiplied by the type 2 diabetes incidence in the total population to determine type 2 diabetes incidence in the group with GDM

^c^RR for developing GDM in intervention vs usual care groups

T2DM, type 2 diabetes mellitus

The annual risk of death was age- and sex-specific for all health states. The all-cause mortality rate among women aged ≥ 40 years with type 2 diabetes was taken from the NDSS, linked to the National Death Index, using data from 2016 [22, 25]. The ‘no type 2 diabetes’ mortality rate was calculated by subtracting type 2 diabetes deaths from the all-cause mortality rate for the Australian female population (2016) [25]. The mortality rate for women aged < 40 years was assumed to be the same for the ‘no type 2 diabetes’ and ‘type 2 diabetes’ health states, as there were very few deaths attributable to type 2 diabetes in this age group. For women aged ≥ 40 years, the mortality rate for the ‘no type 2 diabetes’ health state was derived by subtracting the prevalent type 2 diabetes population and number of annual deaths among women with type 2 diabetes from the total Australian population and annual all-cause mortality rate for each age group. Poisson regression with restricted cubic splines was used to derive single-year age-specific risks for the ‘no type 2 diabetes’ health state.

The NDSS cohort in this study was linked to the Australian Pharmaceutical Benefits Scheme (PBS) to assign diabetes type [3]. The PBS collects information on all prescriptions dispensed in Australia under the scheme, and includes virtually all prescriptions for insulin. We used data from 1 January 2002 to 31 December 2019 for this purpose. Registrants were classified as having type 1 diabetes if they were assigned as having type 1 diabetes by the registering healthcare practitioner, and met any one of the following criteria: (1) less than a year between diagnosis of diabetes and their first prescription for insulin; (2) when date of diagnosis was missing, there was evidence of insulin use at registration on the NDSS and the registrant was < 45 years old at registration; or (3) for those with an age of diagnosis < 30 years (or, if lacking a diagnosis date, registration at age < 45 years) who registered on the NDSS prior to 2002, and whose insulin initiation date on the NDSS was missing, there had to be evidence of ongoing treatment with insulin early in the years for which data from the PBS were available (from 2002). Additionally, type 1 status was assigned to registrants whose original assignment was type 2, but who were < 30 years old at diabetes onset and showed evidence of insulin use within a year, a pattern that is more consistent with type 1 diabetes. An additional requirement for assigning type 1 diabetes was evidence of ongoing treatment with insulin (≥ 2 prescriptions for insulin on the PBS), except when time to end of follow-up/death was < 2 years. Individuals who did not satisfy any of these criteria were classified as having type 2 diabetes. Half-cycle correction was applied to years of life spent in each health state.

### Outcomes of intervention

The lifestyle intervention modelled in this study incorporates structured dietary or physical activity (PA) components, delivered separately or together during early pregnancy, with or without behavioural change, allowing tailoring to meet individual goals and preferences (ESM Table 3) [12]. The intervention effect was modelled for four subgroups (diet only, PA only, diet and PA, diet and/or PA), with the impact on risk of GDM derived from a recently published meta-analysis (Table 1) [12]. The diet and/or PA group, which is the aggregate result for other subgroups, was used in the base case. As clinical trial data for the long-term effect of pregnancy lifestyle intervention on women’s conversion to type 2 diabetes are unavailable, we modelled this parameter in three ways. In the base case, two important assumptions were made: (1) that the elevated risk of developing type 2 diabetes for pregnant women with GDM compared to those without (RR = 9.51) persisted throughout life; and (2) that women who avoided GDM through intervention have the same lifetime risk of type 2 diabetes as those women who did not have GDM in the usual care group. Scenario analyses were then performed to test these two assumptions: scenario 1, in which the elevated risk of developing type 2 diabetes in the GDM group only persisted for 10 years post-birth, then reverted to a level of risk equivalent to the general female population, and scenario 2, in which the RR of conversion to type 2 diabetes for women who avoided GDM through intervention was varied across the range from 1 (equivalent to women who did not develop GDM in the usual care group, as for the base case) to 9.5 (equivalent to if they had GDM, therefore no impact of intervention on risk of type 2 diabetes).

For each intervention subgroup, the following health outcomes were reported: number of cases of GDM and type 2 diabetes, total years of life lived, total years of life lived with type 2 diabetes, and number of NICU/SCN admissions.

### Utility weights

The metric of QALYs adjusts years of life lived by a health utility weight. Individuals in the ‘no type 2 diabetes’ health state were assigned age-specific health utility weights from an Australian cross-sectional study of approximately 3000 individuals in the general population [26]. Individuals in the ‘type 2 diabetes’ health state were assigned a utility weight of 0.785, as reported in a recent systematic review [27]. All utilities were adjusted consistently with their age-specific healthy scores. As type 2 diabetes progresses, both macro- and microvascular complications are common, and are associated with various decreases in the reported health-related quality of life [27]. However, incorporating the costs and consequences of these complications requires numerous modelling assumptions, introduces further uncertainty into the analysis, and is beyond the scope of this model. Therefore, to take a conservative approach, all individuals in the ‘type 2 diabetes’ health state were assigned a health utility weight consistent with uncomplicated type 2 diabetes. This approach, together with the magnitude of utility decrement associated with diabetes, is consistent with other recent economic models of lifestyle interventions to prevent type 2 diabetes [23]. There is no evidence for a decrease in health utility for women with GDM compared to those without [28], and so health utility was not modelled for the GDM health state in this model.

### Intervention design and cost

The base case costing for the intervention was taken from a recent budget impact analysis for Australia [15]. This included the salary of the health coaches delivering the intervention (e.g. registered dietitians, midwives or exercise physiologists), together with salary on-costs (15%) and a fixed cost allowance (20%) for training, facility hire, information technology and administrative support. Based on a midpoint full-time salary from published Australian public sector wage agreements of AU$82,600 (2022 prices), and assuming an average caseload of 500 women per coach per year, the intervention cost was modelled at AU$228 per woman [15]. The costs for discrete intervention subgroups (diet only, PA only, diet and PA) have been published previously [12, 14]. The costs for the diet-only, PA-only, and diet and PA subgroups were derived from a study by Bailey et al [14], who extracted data on intervention design for all studies included in the Teede et al meta-analysis [12], and derived a per patient cost based on profession of practitioner delivering the intervention, patient contact minutes, and whether the intervention was delivered individually or in groups (ESM Table 3). A mean cost for each intervention subgroup was then derived, and this was inflated to 2022 prices for the current study. Interventions with a PA component were costed on the basis of ten women per group [14]. The diet-only, PA-only, and diet and PA intervention groups were mutually exclusive; studies were only assigned to one of these groups in the subgroup meta-analysis. However, the base case incorporated all studies included in these subgroups.

### Antenatal and birth costs

Unit costs for mode of birth, induction of labour and antenatal care for GDM were calculated using the methods described previously [13, 15]. Briefly, costs of antenatal care for GDM were derived from patient pathways developed for a previous cost-effectiveness analysis, incorporating relevant item costs from the Medical Benefits Scheme (MBS), PBS and hospital admissions [13]. The incidence of Caesarean section and/or induction of labour in women with GDM compared with normoglycaemic pregnancies was calculated from the Maternity1000 database comprising mothers who gave birth in Queensland, Australia, between July 2012 and June 2019 (*n* approximately 360,000). All costs are reported in AU$, and were inflated to 2022 prices using the Australian health price index [29].

We tested the impact of incorporating NICU and SCN admission costs by repeating the analysis of cost-effectiveness for each intervention subgroup. Incidence and cost of NICU and SCN admissions for GDM vs normoglycaemic pregnancies were obtained from the Maternity1000 database (ESM Table 4).

### Type 2 diabetes management costs

Type 2 diabetes management costs were taken from two alternative sources and compared. The base case analysis used population-level cost data from the Australian Institute of Health and Welfare Health Expenditure by Burden of Disease Group dataset for 2015–2016 adjusted to 2022 prices (cost derivations are described in ESM Table 5) [8]. This dataset was selected as the primary source of cost data because of the availability of age- and sex-specific costings. The mean cost per prevalent case of type 2 diabetes was derived based on the age-specific rate of disease. This source only provides costs directly attributed to type 2 diabetes, not total healthcare costs among people with type 2 diabetes; thus, in this approach, chronic healthcare costs were only applied to the ‘type 2 diabetes’ health state.

A scenario analysis was also completed using a secondary data source obtained from an Australian micro-costing study (inclusions described in ESM Table 6) [30]. The mean annual cost of ‘newly diagnosed diabetes’ was applied in the model for the first year of diagnosis, after which the mean annual cost for ‘known diabetes’ was applied. While individuals with both type 1 and type 2 diabetes were included in the study, no evidence of cost differences between the diseases was found [30]. Analyses from both the health provider perspective (scenario 3) and societal perspective (scenario 4) were completed using this secondary data source, including direct non-health costs and government income subsidies as described in ESM Table 6.

### Scenario and sensitivity analysis

Scenario analysis was undertaken to explore the impact of altering assumptions around the risk of progression to type 2 diabetes after GDM and lifestyle intervention (scenarios 1 and 2) and type 2 diabetes-related healthcare and societal costs (scenarios 3 and 4), as described in the relevant sections above. Deterministic sensitivity analysis was undertaken to explore the uncertainty surrounding costs (using 95% CI, assuming the SE is 10% of the mean), intervention effect, risk of developing type 2 diabetes after GDM vs normoglycaemic pregnancy and utility weights (published 95% CIs) [5, 12, 26, 27]. Intervention cost was modelled over an extended range to determine the cost at which the intervention was no longer cost-effective. Probabilistic sensitivity analysis was performed for all scenarios (using 10,000 iterations) to account for uncertainty in multiple parameters (costs, health utilities, intervention effect and risk of progression from GDM to type 2 diabetes), sampling from distributions as listed in Table 1 and ESM Tables 3–5.

## Results

### Base case

Over the lifetime horizon, the model projected a 10% reduction in the number of women diagnosed with type 2 diabetes (−8248 cases; 95% CI −11,419, -4554, ) and over 45,000 fewer years of life lived with type 2 diabetes through routine implementation of the lifestyle intervention programme compared with current usual care (Table 2). The total net incremental cost of the intervention was approximately AU$70 million. The total net incremental healthcare cost savings from a reduction in pregnancy costs and type 2 diabetes management costs was approximately AU$85 million. The intervention was dominant (cost-saving) compared with usual care from a healthcare perspective, and gave an ROI of AU$1.22 (95% CI 0.53, 2.13) per dollar invested. Inclusion of NICU/SCN costs introduced further healthcare cost savings of approximately AU$24 million, and an ROI of AU$1.57 (95% CI 0.75, 2.58). The PA-only intervention subgroup was extendedly dominant over other interventions, although confidence intervals here were larger than in the base case (ROI AU$1.63; 95% CI 0.50, 3.00). When NICU/SCN costs were included, all intervention groups were dominant over usual care, although uncertainty around this cost parameter resulted in wider confidence intervals for the incremental cost and ROI outcomes (Table 2).

**Table 2:** Results comparing usual care with routine structured diet and PA intervention categories

		Incremental change due to intervention-by-intervention subgroup			
Outcome	Usual care	Base case (diet and/or PA)	Diet only	PA only	Diet and PA
Excluding NICU/SCN					
Health outcomes					
Cases of GDM	37,957	−12,526 (−17,341, −6923)	−19,905 (−21,508, −1350)	−14,424 (−21,585, −5171)	−9489 (−18,035, 1514)
Cases of type 2 diabetes	85,219	−8248 (−11,419, −4554)	−8498 (−14,158, −900)	−9498 (−14,199, −3419)	−6249 (−11,863, 1001)
YLL^a^	5,773,306	2576	2654	2966	1951
YLL with type 2 diabetes^a^	328,625	−45,739	−47,125	−52,669	−34,651
QALYs^a^	5,123,076	10,748	11,074	12,376	8142
Incremental QALYs per woman		0.035 (0.020, 0.050)	0.036 (0.004, 0.061)	0.041 (0.015, 0.061)	0.027 (−0.004, 0.051)
Costs (AU$)					
Intervention cost	0	69,904,575	54,142,597	60,265,867	69,934,187
Pregnancy costs	3,142,840,956	−25,187,141	−25,950,388	−29,003,375	−19,081,168
Type 2 diabetes management	425,992,395	−60,108,916	−61,930,398	−69,216,327	−45,537,057
Total healthcare costs^a^	3,568,833,351	−15,391,482	−33,738,190	−37,953,836	5,315,962
Incremental cost per woman		−50 (−230, 110)	−110 (−373, 153)	−124 (−373, 102)	17 (−229, 267)
ICER		Dominant	Dominant	Dominant	653
PSA iterations within cost-saving quadrant (%)		70.5	81.6	85.7	47.9
ROI ratio		1.22 (0.53, 2.13)	1.62 (0.16, 3.22)	1.63 (0.50, 3.00)	0.92 (−0.14, 2.05)
Including NICU/SCN					
Health outcomes					
NICU/SCN admissions	71,847	−1704 (−2359, −941)	−1755 (−2925, −184)	−1962 (−2936, −703)	−1291 (−2453, 206)
Costs (AU$)					
NICU/SCN costs	1,218,144,257	−24,264,341	−24,999,624	−27,940,757	−18,382,077
Total healthcare costs^a^	4,786,977,608	−39,655,823	−58,737,814	−65,894,592	−13,066,114
Incremental cost per woman		−130 (−337, 57)	−192 (−629, 132)	−216 (−496, 63)	−43 (−341, 276)
ICER		Dominant	Dominant	Dominant	Dominant
PSA iterations within cost-saving quadrant (%)		90.7	90.3	94.2	66.1
ROI ratio		1.57 (0.75, 2.58)	2.08 (0.20, 4.02)	2.09 (0.69, 3.64)	1.19 (−0.17, 2.55)

Values are means, with 95% CI in parentheses where applicable

^a^Discounted at 5% discounting rate

PSA, probabilistic sensitivity analysis; YLL, years of life lived

### Sensitivity and scenario analysis

**Fig. 2:**
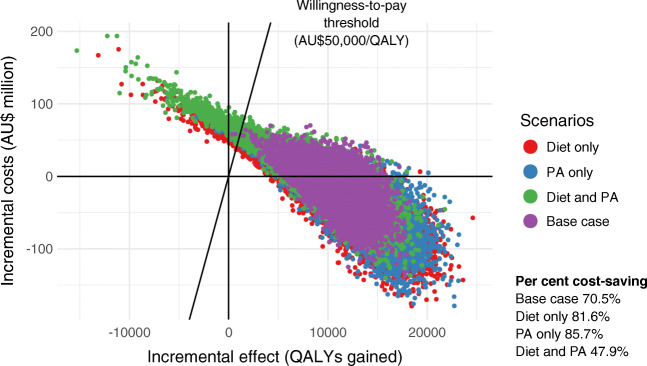
Results of the probabilistic sensitivity analysis for base case and intervention categories shown on the cost-effectiveness plane (AU$ million). The percentage of iterations that were cost-saving for each intervention subgroup is indicated at the bottom right

A one-way sensitivity analysis showed that the model results were most sensitive to uncertainty around the RR of developing GDM and birth costs (GDM and normoglycaemic pregnancies). While the ranges explored for these parameters produced results that were not cost-saving, they remained cost-effective, as no ICERs returned by the model were greater than AU$1200/QALY (ESM Fig. 1). The intervention was cost-effective if its cost remained below AU$2030 per participant (ESM Fig. 2). Probabilistic sensitivity analysis for the base case showed that 70.5% of the simulations were cost-saving (Fig. 2), while for the diet-only, PA-only and diet and PA subgroups, 81.6%, 85.7% and 47.9% of simulations, respectively, were cost-saving.

**Fig. 3:**
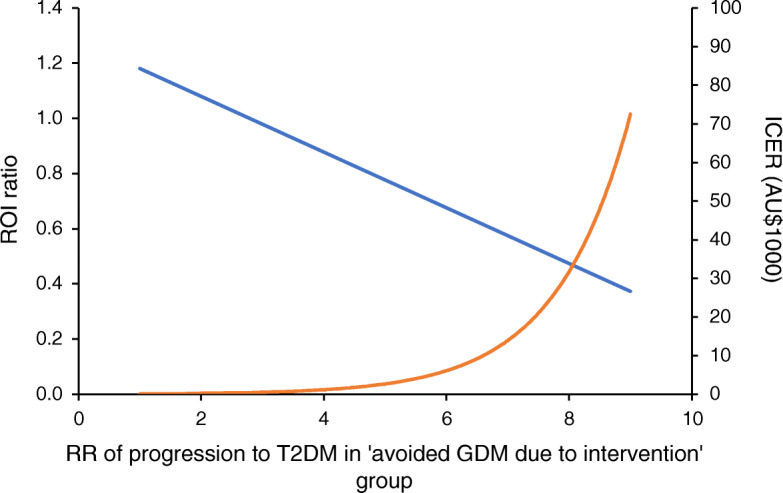
Sensitivity analysis showing the impact of variation in the RR of developing type 2 diabetes in the group who avoided GDM through intervention (compared to the group without GDM under usual care) on the ROI ratio (blue line) and ICER (orange line) (scenario 2). T2DM, type 2 diabetes mellitus

In scenario 1, the ROI decreased to 0.72 (95% CI 0.17, 1.44), although the intervention was still highly cost-effective, with an associated ICER of AU$5409/QALY (Table 3). When varying the RR of developing type 2 diabetes in the group that avoided GDM through intervention (scenario 2), an RR of 2.71 resulted in an ROI of 1 (ICER AU$0/QALY), and an RR of 8.62 resulted in an ICER of AU$50,000/QALY (Fig. 3); thus, RRs lower than these would be cost-saving and cost-effective, respectively. Scenarios 3 and 4 showed the cost-effectiveness of the intervention to be dominant over usual care, with ROIs higher than for the base case: 1.65 (95% CI 0.80, 2.71) and 4.37 (95% CI 2.33, 6.63), respectively (Table 3 and ESM Table 7).

**Table 3:** Health outcomes, ICER and ROI ratios for the base case and scenarios 1, 3 and 4

Scenario	Value
Base case	
Number of T2DM cases prevented	8248 (4554, 11,419)
Incremental QALYs per woman	0.035 (0.020, 0.050)
Incremental costs (AU$) per woman	−50 (−230, 110)
ICER (AU$/QALY)	Dominant
PSA iterations within cost-saving quadrant	70.5%
ROI ratio	1.22 (0.54, 2.07)
Scenario 1	
Number of T2DM cases prevented	1002 (554, 1388)
Incremental QALYs per woman	0.012 (0.006, 0.017)
Incremental costs (AU$) per woman	63 (−96, 196)
ICER (AU$/QALY)	5409
PSA iterations within cost-saving quadrant	19.5%
ROI ratio	0.72 (0.17, 1.44)
Scenario 3	
Incremental costs (AU$) per woman	−148 (−365, 47)
ICER (AU$/QALY)	Dominant
PSA iterations within cost-saving quadrant	92.6%
ROI ratio	1.65 (0.80, 2.71)
Scenario 4	
Incremental costs (AU$) per woman	−770 (−1190, −331)
ICER (AU$/QALY)	Dominant
PSA iterations within cost-saving quadrant	99.9%
ROI ratio	4.37 (2.33, 6.63)

The base case represents the healthcare perspective (AU$/QALY) and used the diet and/or PA intervention group and excluded NICU/SCN costs

Scenario 1 considers the elevated risk of type 2 diabetes limited to 10 years post-birth (AU$/QALY)

Scenario 3 comprises a micro-costing for type 2 diabetes from a healthcare perspective (AU$/QALY)

Scenario 4 comprises a micro-costing for type 2 diabetes from a societal perspective (AU$/QALY)

The ROI and ICER for scenario 2 are shown in Fig. 3

PSA, probabilistic sensitivity analysis; T2DM, type 2 diabetes mellitus

## Discussion

To our knowledge, this is the first study to use decision analysis with life table modelling to estimate the long-term economic outcomes of a lifestyle intervention during pregnancy that aimed to reduce the incidence of GDM and subsequent development of type 2 diabetes. We demonstrate that a structured diet and PA intervention, when offered to all women during pregnancy, is highly cost-effective in all scenarios explored, from both healthcare and societal perspectives. Three of four intervention groups were cost-saving from a healthcare perspective compared with usual care, with all groups becoming cost-saving with the inclusion of NICU/SCN costs. While limiting the elevated risk of progression to type 2 diabetes to the first 10 years after pregnancy (most conservative scenario) reduced the number of type 2 diabetes cases prevented (from 8000 to 1000) and the associated health cost savings, the ICER returned by this scenario was still highly cost-effective. Therefore, given our conservative assumptions, it is likely that the lifestyle intervention modelled in this study will provide significant economic and health benefits. These findings highlight the potential benefits of routine implementation of a lifestyle intervention during pregnancy on Australian women's health outcomes and quality of life across their lifespan.

Previous studies of the cost-effectiveness of interventions to reduce GDM incidence have focused almost exclusively on clinical trial data from the intrapartum period alone [13, 14, 28]. One long-term study failed to detect an improvement in QALYs between the intervention and control groups after 7 years of follow-up; however, no cases of type 2 diabetes were detected in either group after 7 years, suggesting that the sample was not representative of Australia’s population [31]. Studies that have taken a lifetime perspective have not accounted for the impact of the intervention on long-term maternal morbidity, or have focused on interventions used in the treatment of GDM rather than prevention [18, 32, 33]. Model-based evaluations such as ours are required to assess the impact of GDM prevention, to account for events that affect costs or outcomes over the longer term [34].

Several studies have modelled the cost-effectiveness of lifestyle interventions to prevent type 2 diabetes over a lifetime horizon, both in postpartum populations with a history of GDM [23] and in general populations [34]; these informed the design of this model. Intensive lifestyle interventions incorporating both diet and PA components have been shown to be cost-effective from a healthcare perspective, although interventions examined in other lifetime modelling studies are of greater intensity (number and frequency of sessions) and have a much higher cost than ours [23, 34, 35]. Importantly, however, these interventions aimed to facilitate weight loss, rather than to prevent weight gain like ours. These studies also targeted populations at increased risk for developing type 2 diabetes, and consequently reported greater effectiveness in reducing the lifetime incidence of type 2 diabetes. Despite this, our model returned greater cost savings from intervention, probably due to a combination of the antenatal period presenting a critical prevention window within which to implement lifestyle change [12] and the additional significant health cost savings occurring during pregnancy and birth.

The strengths of this study include the flexible approach to intervention design and costing, which allows for realistic real-world implementation across diverse health services. Importantly, fixed infrastructure expenses and training of professionals delivering the intervention were included in the costing; these components are frequently overlooked in other lifestyle intervention economic models [34]. Real-world data was also used extensively in the modelling, with the parameters for GDM and type 2 diabetes incidence, mortality and costs derived directly from routine data collected by national and state government agencies. The effect of intervention was taken from a meta-analysis of 117 clinical trials with a wide geographic distribution of samples. Therefore, the results are highly representative of the population of Australian mothers. However, care should be taken when generalising the results to other populations and healthcare systems, where the incidence of GDM and efficacy of lifestyle interventions during pregnancy may differ.

There were several limitations to the study design. We limited the model scope to health conditions and adverse health events that could be confidently costed (Caesarean section birth, induction of labour, admission to NICU or SCN, and clinical management of GDM and type 2 diabetes). Recent evidence suggests that GDM may be associated with other long-term maternal health outcomes, such as an elevated risk of developing cardiovascular events [36], which we have not accounted for in our model. Moreover, due to a lack of evidence, we have not accounted for the health-related quality of life benefits for women arising from reduced body weight, independent of type 2 diabetes diagnosis [37], or the impact of GDM prevention on long-term health outcomes for offspring [31, 38]. Due to a paucity of data, we assumed that health utility outcomes in postpartum women were consistent with wider population-based values, which may not be realistic. More research is required to establish health utility norms for pregnant and postpartum women. Another limitation is that the mean age of individuals in the micro-costing data obtained from the Australian Diabetes, Obesity and Lifestyle (AusDiab) study (ESM Table 6) [30] was 60 years, which may not capture the actual cost of disease for the target population of our model (women aged 15–49 years after pregnancy). Furthermore, the societal perspective in this study used government support payments as a proxy for lost productivity, which probably underestimates lost earnings as support payments are lower than the minimum wage. We also did not incorporate the significantly elevated health costs incurred during later pregnancies following a diagnosis of GDM, despite the increased risk of recurrent GDM and/or type 2 diabetes during these pregnancies, as the impact of the intervention on outcomes in subsequent pregnancies is unknown. This approach is conservative, as pregnancies with type 2 diabetes are high risk, and inclusion of these costs would increase the cost savings returned in the model [39]. Similarly, to be conservative, type 2 diabetes utility weights were estimated using only values for uncomplicated diabetes, as there is a significant utility reduction imposed by many diabetes complications [27].

A fundamental assumption of our model was that women for whom GDM was avoided via the lifestyle intervention would have the same long-term risk of developing type 2 diabetes as women who did not develop GDM in the usual care group. Data on the long-term incidence of type 2 diabetes following lifestyle interventions for GDM are limited [33, 34]; we therefore modelled a variety of scenarios to estimate the effects of this uncertainty. Notably, only a modest decrease in the RR of developing type 2 diabetes for women who avoid GDM through intervention (compared to those without GDM in the usual care group) from 9.5 to 8.6 would be required for the intervention to reach an acceptable cost-effectiveness threshold of AU$50,000/QALY. It is likely that the RR is much lower, although more research is needed on the long-term effects of pregnancy lifestyle interventions. Until such data are available, modelling studies such as that presented here can fill important information voids, provided decision-makers give adequate consideration to the uncertainty inherent in the model. This issue is further complicated by the controversy surrounding diagnostic criteria for GDM, and the impact that these have on documented incidence and treatment outcomes at the population level. These criteria have been described as too sensitive, increase the prevalence of GDM [40, 41], and are not based on risk of type 2 diabetes progression like the older GDM criteria were. We minimised the effects of diagnostic criteria on model parameters by using population data from a time point (2016) consistent with the data sources used in the meta-analyses that produced the treatment effect parameters applied in the model. Application of the IADPSG criteria may change the intervention's effectiveness and the costs associated with GDM from those used in this model.

In conclusion, routine implementation of a structured lifestyle intervention during pregnancy was highly cost-effective from healthcare and societal perspectives, for all explored scenarios. The reduction in healthcare costs associated with managing adverse events during pregnancy and maternal morbidity from type 2 diabetes over the lifetime horizon offset the costs of intervention delivery, despite conservative assumptions. Significant health benefits were also found, increasing both quality and quantity of life for women receiving the intervention.

## Supplementary Information

ESM 1 (PDF 358 kb)

## Data Availability

The data used in this study were sourced from the National Diabetes Services Scheme (NDSS) and the Maternity1000 database. The NDSS is an initiative of the Australian Government administered by Diabetes Australia. Maternity1000 is administered by the Australian Institute of Health and Welfare (AIHW), with records of all included individuals linked to the Queensland Hospital admitted patient data collection, non-admitted patient data collection, deaths registry, emergency department information system and hospital and health service funding and costing unit records between 1 July 2012 and 30 June 2019. Use of NDSS data was approved by the Alfred Hospital Ethics Committee (project number 15/15) (Melbourne, VIC, Australia) and the AIHW Ethics Committee (EO 2015/1/148) (Canberra, ACT, Australia). Use of Maternity1000 data was approved by the Townsville Hospital and Health Service Human Research Ethics Committee (HREC/16/QTHS/223) and the AIHW Ethics Committee (EO2017-1-338). The datasets generated and/or analysed during the current study are available from the corresponding author on reasonable request.

## Abbreviations

GDM: Gestational diabetes mellitus
IADPSG: International Association of Diabetes in Pregnancy Study Group
ICER: Incremental cost-effectiveness ratio
NDSS: National Diabetes Services Scheme
NICU: Neonatal intensive care unit
PA: Physical activity
PBS: Pharmaceutical Benefits Scheme
QALY: Quality-adjusted life year
ROI: Return on investment
SCN: Special care nursery
