# Supplementary material for: Long-term cost-effectiveness of implementing a lifestyle intervention during pregnancy to reduce the incidence of gestational diabetes and type 2 diabetes
Source: Diabetologia. 2023 Mar 18;66(7):1223–34. doi: 10.1007/s00125-023-05897-5 (PMC10244289; doi:10.1007/s00125-023-05897-5)
Supplement: Supplementary file 1 — (PDF 358 kb) [file 125_2023_5897_MOESM1_ESM.pdf]

Electronic Supplementary Materials

Long term cost-effectiveness of implementing a lifestyle intervention during pregnancy to reduce the incidence of gestational diabetes and type 2 diabetes

Authors: Melanie Lloyd, Jedidiah Morton, Helena Teede, Clara Marquina, Dina Abushanab, Dianna J. Magliano, Emily J. Callander, Zanfina Ademi.

Table of Contents

ESM Table 1. Population characteristics for women giving birth in Australian hospitals (2015-16) 3

ESM Table 2. Age-specific mortality rates and Type 2 Diabetes incidence (Australian females, 2016). 4

ESM Table 3. Health cost parameters (AU$, converted to 2022 prices) 6

ESM Table 4. Neonatal Intensive Care (NICU) and Special Care Nursery (SCN) admissions and costs. 7

ESM Table 5. Type 2 diabetes direct health costs per person – population level data (AU$, converted to 2022 prices). 7

ESM Table 6. Annual Type 2 diabetes direct and indirect health costs per person – derived from bottom-up microcosting used in Scenarios 3 and 4 (AU$, converted to 2022 prices) 8

ESM Table 7. Total and incremental type 2 diabetes cost outputs from Scenarios 3 and 4 (AU$, 2022 prices). 8

ESM Figure 1. One-way sensitivity analysis showing uncertainty around key parameters in the model (base case). 9

ESM Figure 2. One-way sensitivity analysis exploring impact of varying intervention cost on Incremental Cost-Effectiveness Ratio (ICER) 9

References 10

ESM Table 1. Population characteristics for women giving birth in Australian hospitals (2015-16)

	Diagnosed with GDM		Pre-existing diabetes	
**Total (*n*)***	**(*n*)**	**(%)**	**(%)**	**Source**
7648	437	5.7	0.5	AIHW[1]
38,189	3068	8.0	0.8	
83,871	8954	10.7	0.8	
109,957	13,935	12.7	0.9	
56662	8850	15.6	1.3	
12,390	2506	20.2	1.7	
848	207	24.4	1.7	
**308,593**	**37,957**	**12.3**	**1.0**	

*Women aged 15-49 based on ICD-AM-10 diagnosis.[2]

ESM Table 2. Age-specific mortality rates and Type 2 Diabetes incidence (Australian females, 2016).

	NO TYPE 2 DIABETES GROUP ALL CAUSE DEATH	TYPE 2 DIABETES GROUP ALL CAUSE DEATH[3]	TYPE 2 DIABETES INCIDENCE[3]
Age (years)	Mortality rate	Mortality rate	
15	0.0001653	0.000165	0.0000454459
16	0.0001779	0.000178	0.0000626415
17	0.0001901	0.000190	0.00007151
18	0.0002023	0.000202	0.00016414
19	0.0002146	0.000215	0.00019772
20	0.0002273	0.000227	0.00015920
21	0.0002407	0.000241	0.00016863
22	0.0002550	0.000255	0.00022649
23	0.0002703	0.000270	0.00026408
24	0.0002871	0.000287	0.00035853
25	0.0003054	0.000305	0.00030032
26	0.0003256	0.000326	0.00033558
27	0.0003478	0.000348	0.00049928
28	0.0003724	0.000372	0.00046444
29	0.0003995	0.000400	0.00056583
30	0.0004294	0.000429	0.00059836
31	0.0004624	0.000462	0.00065850
32	0.0004987	0.000499	0.00082864
33	0.0005384	0.000538	0.00077155
34	0.0005820	0.000582	0.00112725
35	0.0006295	0.000629	0.00109360
36	0.0006813	0.000681	0.00110630
37	0.0007375	0.000738	0.00114035
38	0.0007985	0.000798	0.00118526
39	0.0008644	0.000864	0.00120249
40	0.000774	0.002252	0.00147701
41	0.000831	0.002252	0.00176381
42	0.000892	0.003604	0.00166092
43	0.000959	0.004714	0.00171456
44	0.00103	0.004032	0.00187613
45	0.001108	0.002484	0.00216875
46	0.001192	0.004085	0.00242707
47	0.001285	0.002807	0.00271507
48	0.001386	0.004619	0.00242336
49	0.001498	0.003633	0.00323766
50	0.001619	0.004233	0.00297445
51	0.001752	0.004484	0.00381154
52	0.001897	0.003316	0.00357596
53	0.002053	0.006205	0.00408272
54	0.002222	0.005090	0.00419321

55	0.002402	0.006577	0.00445309
56	0.002594	0.005816	0.00460500
57	0.002798	0.005914	0.00518559
58	0.003014	0.006809	0.00492529
59	0.003246	0.007901	0.00524348
60	0.003497	0.007536	0.00504630
61	0.00377	0.007027	0.00596857
62	0.00407	0.006334	0.00666360
63	0.004403	0.010206	0.00616856
64	0.004774	0.010348	0.00613777
65	0.005192	0.011315	0.00653793
66	0.005665	0.012483	0.00642023
67	0.006203	0.012814	0.00650452
68	0.006816	0.011020	0.00706555
69	0.007517	0.016726	0.00720502
70	0.008323	0.017713	0.00772435
71	0.009254	0.018448	0.00759395
72	0.010333	0.020508	0.00760197
73	0.011589	0.025182	0.00811246
74	0.013054	0.023651	0.00773031
75	0.014767	0.031685	0.00806784
76	0.016774	0.033450	0.00874526
77	0.019131	0.029014	0.00853819
78	0.021907	0.034043	0.00834658
79	0.025182	0.042690	0.00837954
80	0.029055	0.046470	0.00791778
81	0.033636	0.053928	0.00849403
82	0.039001	0.061352	0.00804421
83	0.045199	0.058670	0.00670946
84	0.05225	0.076283	0.00573947
85	0.060122	0.075751	0.00609273

Notes. The ‘No type 2 diabetes’ mortality rate was calculated by subtracting type 2 diabetes deaths from the all-cause mortality rate for the Australian female population (2016)[4]. The age-specific ‘Type 2 diabetes’ mortality rate was taken from the National Diabetes Services Scheme (NDSS) dataset.[3]

ESM Table 3. Health cost parameters (AU$, converted to 2022 prices)

	Range*				Source
	**Mean**	**Lower**	**Upper**	**Distribution**	
Antenatal GDM management costs	1113	895	1331	Gamma	Bailey et al.[5]
Birth cost GDM pregnancy	10,899	8,763	13,035	Gamma	Bailey et al.[5], Maternity1000
Birth cost normoglycemic pregnancy	10,002	8,041	11,962	Gamma	Bailey et al.[5], Maternity1000
Lifestyle intervention cost (per woman)					
• Base case (Diet AND/OR PA)	228	183	273	Gamma	Lloyd et al.[6]
• Diet only	177	142	211	Gamma	Bailey et al.[5]
• PA only	197	158	235	Gamma	Bailey et al.[5]
• Diet AND PA	228	183	273	Gamma	Bailey et al.[5]
Type 2 diabetes management cost	Age-specific – See ESM Table 5.			Gamma	AIHW[7]

*Range used in deterministic sensitivity analysis.

Abbreviations: AU$: Australian dollars; GDM: gestational diabetes; PA: physical activity.

ESM Table 4. Neonatal Intensive Care (NICU) and Special Care Nursery (SCN) admissions and costs.

	NICU	SCN
% neonates from GDM pregnancies	2.3	33.1
% neonates from normoglycemic pregnancies	2.1	19.8
Average admission cost (AU$, converted to 2022 prices)	53,319	13,398

Source: Maternity1000 dataset.

Abbreviations: GDM: gestational diabetes; NICU: neonatal intensive care unit; SCN: special care nursery.

ESM Table 5. Type 2 diabetes direct health costs per person – population level data (AU$, converted to 2022 prices).

		Range*	
Age group	Cost / female / year	Lower	Upper
15-19	45,713	36,723	54,672
20-24	18,075	14,532	21,618
25-29	10,521	8459	12,583
30-34	5404	4345	6463
35-39	2697	2168	3226
40-44	1658	1333	1984
45-49	1233	992	1475
50-54	1154	928	1380
55-59	1103	886	1319
60-64	1078	867	1289
65-69	1088	875	1302
70-74	1033	830	1235
75-79	1091	878	1305
80-84	1103	887	1320

Source: AIHW Disease Expenditure in Australia 2015-16[7]

*Range used in deterministic sensitivity analysis.
Note: This dataset captures direct health expenditure related to hospital admissions; Emergency Department presentations; hospital outpatient services; medical, dental and health practitioner services funded under the MBS or Department of Veterans Affairs; and pharmaceuticals. Expenditure was mapped to specific conditions via a mixture of top-down and bottom-up costing approaches, with the allocation of expenditure driven by primary diagnosis classification (though secondary diagnoses and comorbidities were captured in modelling).

ESM Table 6. Annual Type 2 diabetes direct and indirect health costs per person – derived from bottom-up microcosting used in Scenarios 3 and 4 (AU$, converted to 2022 prices)

	Direct healthcare costs	Direct non-healthcare costs	Total direct costs	Government subsidies
Normal glycaemic tolerance	1955	611	2568	4545
New Diabetes	2814	836	3650	6285
Known Diabetes	4064	1873	5936	7794

Source: Lee et al. Diabetes Research and Clinical Practice 2013;99:385-390.[9] (2004-05 prices)

Note: Cost definitions - Direct health costs (ambulatory services, hospitalizations, prescription medications and medically related consumables), direct non-health costs (transport to hospital, supported accommodation and nursing home fees, home-based supportive services and special dietary requirements) and cost of government income subsidies (as a proxy for lost productivity; including aged, disability and veteran pensions, mobility or sickness allowances and unemployment benefits) for individuals with diabetes compared to a normoglycemic control population.

ESM Table 7. Total and incremental type 2 diabetes cost outputs from Scenarios 3 and 4 (AU$, 2022 prices).

	Control	Intervention	Incremental
Healthcare perspective			
Type 2 diabetes direct health costs	11,952,231,824	11,862,330,950	-89,900,874
Total healthcare costs	15,095,072,781	15,049,889,340	-45,183,441
Societal perspective			
Type 2 diabetes direct health costs	11,952,231,824	11,862,330,950	-89,900,874
Direct non-healthcare type 2 diabetes costs	3,924,160,924	3,869,766,485	-54,394,439
Type 2 diabetes-related Government subsidies	27,256,825,693	27,121,061,664	-135,764,030
Total costs	46,276,059,398	46,040,717,489	-235,341,909

ESM Figure 1. One-way sensitivity analysis showing uncertainty around key parameters in the model (base case).

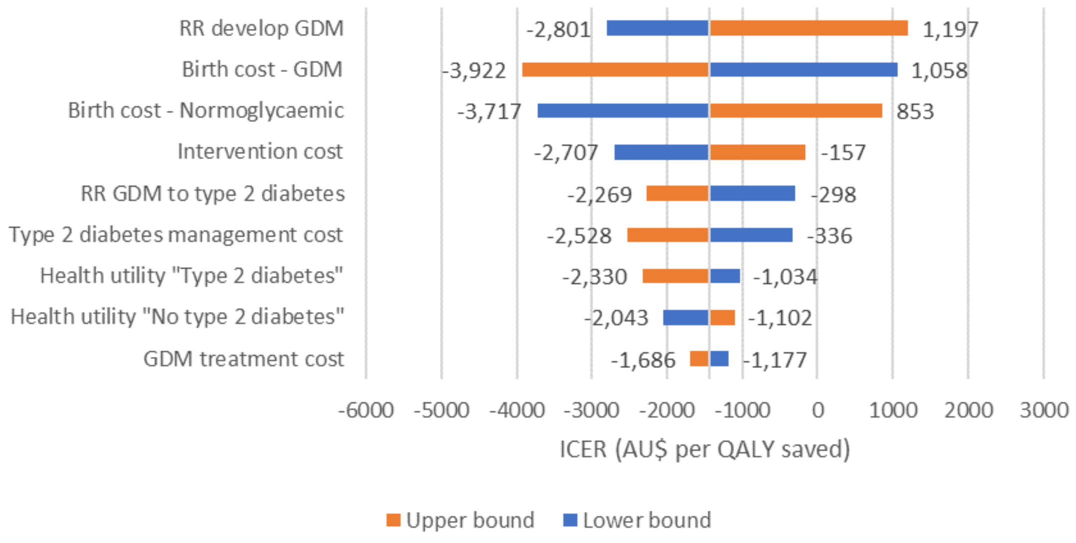

Abbreviations: GDM: Gestational diabetes; ICER: Incremental cost effectiveness ratio; RR: Relative risk.

ESM Figure 2. One-way sensitivity analysis exploring impact of varying intervention cost on Incremental Cost-Effectiveness Ratio (ICER)

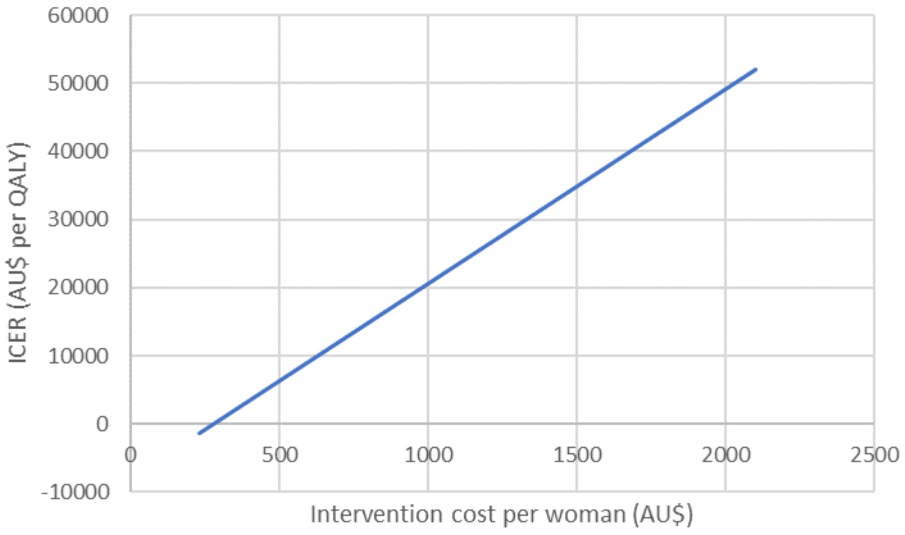

Abbreviations: ICER: Incremental cost effectiveness ratio; QALY: Quality-adjusted life year.

References

1. Australian Institute of Health and Welfare. Data Tables: Australia's Mothers and Babies 2019. [cited: 24 November 2022]. Available from: https://www.aihw.gov.au/reports/mothers-babies/australias-mothers-babies/data

2. Australian Institute of Health and Welfare. Gestational Diabetes. 2018. [cited: 24 November 2022]. Available from: https://www.aihw.gov.au/reports/diabetes/diabetes-compendium/contents/how-many-australians-have-diabetes/gestational-diabetes

3. Morton JI, Lazzarini PA, Shaw JE, Magliano DJ. Trends in the Incidence of Hospitalization for Major Diabetes-Related Complications in People With Type 1 and Type 2 Diabetes in Australia, 2010–2019. Diabetes Care. 2022;45(4):789-97.

4. Australian Institute of Health and Welfare. General Record of Incidence of Mortality (GRIM) books, Data visualisation [Internet]. [cited 24 November 2022]. Available from: https://www.aihw.gov.au/reports/life-expectancy-deaths/grim-books/contents/grim-excelworkbooks

5. Bailey C, Skouteris H, Harrison CL, Hill B, Thangaratinam S, Teede H, et al. A Comparison of the Cost-Effectiveness of Lifestyle Interventions in Pregnancy. Value in Health. 2021;25(2):194-202.

6. Lloyd M TH, Bailey C, Callander EJ, Ademi Z. Projected return on investment from implementation of a lifestyle intervention to reduce adverse pregnancy outcomes. JAMA Network Open. 2022;5(9):e2230683.

7. Australian Institute of Health and Welfare. Disease Expenditure in Australia 2015-16. 2019. [cited: 24 November 2022]. https://www.aihw.gov.au/reports/health-welfare-expenditure/disease-expenditure-australia-2015-16/contents/about

8. Teede HJ, Bailey C, Moran LJ, Khomami MB, Enticott J, Ranasinha S, et al. Association of Antenatal Diet and Physical Activity–Based Interventions With Gestational Weight Gain and Pregnancy Outcomes: A Systematic Review and Meta-analysis. JAMA Internal Medicine. 2022;182(2):106-114.

9. Lee CMY, Colagiuri R, Magliano DJ, Cameron AJ, Shaw J, Zimmet P, et al. The cost of diabetes in adults in Australia. Diabetes Research and Clinical Practice. 2013;99(3):385-90.
